# Differences in the level of physical fitness and mobility among older women with osteoporosis and healthy women—cross-sectional study

**DOI:** 10.1038/s41598-021-93483-3

**Published:** 2021-07-09

**Authors:** Anna Szczygielska Babiuch, Katarzyna Oestervemb, Anna Lipińska, Magdalena Lipińska Stańczak, Magdalena Cholewa, Kamila Makulec, Kinga Nowakowska, Magdalena Hagner Derengowska

**Affiliations:** 1 Cracow University of Health Promotion, ul. Grottgera 1, 30-035 Cracow, Poland; 2 Day Rehabilitation Department, NZOZ PASTERNIK, Modlniczka, Poland; 3 Private Rehabilitation Office Fizjoactive, Kutno, Poland; 4 The Jan Kochanowski University (JKU) in Kielce, Public Health, Physiotherapy, Kielce, Poland; 5 Andrzej Frycz Modrzewski Krakow University, Cracow, Poland; 6 Department of Physical Culture, University Nicolaus Copernicus, Toruń, Poland; 7 Private Physiotherapy Practice “Rehamedicina”, Cracow, Poland

**Keywords:** Medical research, Risk factors, Health occupations, Orthopaedics, Diseases, Rheumatic diseases, Health care, Geriatrics, Therapeutics

## Abstract

The main aim of the study was to assess the risk of falls, and physical fitness in the group of women aged 60 to 65 years of age suffering from an identified osteoporosis in comparison to a similar group of healthy women. The main question was: What is the level of physical fitness and risk of fall among women with osteoporosis compared to healthy women? The research included 262 women aged 60 to 65 of age: 135 with osteoporosis and 127 healthy ones, living in the Małopolskie and the Świętokrzyskie Provinces of Poland. To assess the level of physical fitness, the Senior Fitness Test (SFT) was used, while the Tinetti POMA (Performance Oriented Mobility Assessment) and Timed Up&Go test (TUG) were used to asses the risk of fall. Significant statistical differences in average results of physical fitness assessment were noticed as regards the following aspects: flexibility of the lower body part p < 0.001; flexibility of the upper body part p < 0.001. Essential differences were demonstrated in assessing the risk of falling with p < 0.01. Women with osteoporosis are marked by a lower physical fitness than healthy women. A higher percentage of great and serious risk of fall was demonstrated among women with osteoporosis.

## Introduction

Osteoporosis is a disease that affects the whole man's skeleton system, and it is marked, first of all, by the reduced mechanical strength of the skeleton, which results in increasing the risk of fractures. Those fractures are also called the osteoporotic fractures. Often the disease develops without any symptoms, and the first symptoms appear only when the disease is considerably advanced. This is a serious problem, both medical, and economic one^1^. Treatment strategies are aimed at increasing the processes of osteogenesis^2^.

Osteoporotic fractures may be the result of primary osteoporosis (associated with menopause and aging) and secondary osteoporosis (as a result of various diseases and taking medications that negatively affect bones, e.g. glucocorticoids).

All those elements cause increased risk of falls and fractures among persons aged 60 plus^3^. The growing number of osteoporotic fractures relates to the periods of early and late senility, and the number of older and older senior men and women. The problem is also connected with the rising costs of treatment of fractures themselves and their complications^4^.

Balance disorders among senior women cause, first of all, limitations in their full social life functioning. Striving after improvement of coordination ability and balance aims, predominantly, at the improvement of such women's mobility and an increase in their independence in daily life, and also at reducing the risk of falls and other injuries^5^. Functional fitness plays a very considerable role both in prevention activities, and after a fall. In the case of women suffering from osteoporosis, an appropriate level of physical fitness is also connected with the possibility of undertaking physical effort indispensable for stimulating the osteogenesis processes. Activities involving physical movement influence significantly the improvement of motor coordination and, through that, constitute a specific prophylaxis of such body injuries, like stumbling or falling^6, 7^. Decrease of physical activity, especially among women with osteoporosis, constitutes an independent death risk factor, and may lead to increasing the death rate among senior citizens^8^.

Studies to date have only focused on the risk of fracture among women with osteoporosis, or the risk of fracture associated with the possibility of falling. However, there are no studies on the assessment of the risk of falling in relation to the level of physical fitness, the level of which is one of the most important determinants of the risk of falling. Therefore, it seems justified, both from the point of view of treatment and prophylaxis, to investigate this relationship. Additionally, it seems justified to compare the parameters tested with a control group of healthy women to see if osteoporosis itself has significant influence on them. The goal of the research work was to assess the risk of falls, and physical fitness in the group of women aged 60 to 65 years of age suffering from an identified osteoporosis in comparison to a similar group of healthy women. The questions that study should solve was:

1. What is the level of physical fitness in women with osteoporosis as assessed by means of the Senior Fitness Test (SFT) when compared to healthy women?

2. What risk of fall do osteoporosis women demonstrate as compared to healthy women?

3. What essential correlations do exist between SFT results, and the fall risk tests results?

## Research material and methods

All methods were carried out in accordance with relevant guidelines and regulations by the approval of the Bioethical Commission at the District Medical Chamber in Krakow (consent no. 140/KBL/OIL/2013). All authors confirm that all experimental protocols were approved by that Bioethics Committee.

The research included 262 women, aged 60 to 65 years of age, both suffering from osteoporosis, and healthy ones. The participants were divided into two groups: a group with osteoporosis (GO, N = 135), and a group of healthy women (GH, N = 127). The average age of the GO participants was 63 ± 1.8 years, while that in the GH group amounted to 62 ± 1.9 years. The groups were homogeneous in terms of age. Assignment to particular groups was done on the basis of densitometric bone examination. Densitometric studies were performed with the LUNAR GE DPX PRO apparatus (production year 2010) among women tested in Kraków and Wieliczka, and with the LUNAR GE PRODIGY ADVANCE apparatus (production year 2008). Women with *T*-score results equalling, or lower than − 2.5 SD qualified for the osteoporosis group, while patients with results exceeding − 2.5 SD for the healthy group. The examined women were patients of different osteoporosis treatment and rehabilitation clinics in the cities of Cracow, Wieliczka, and Kielce (Poland), as well as women attending the Third University of the Third Age at the Jagiellonian University in Krakow. Women were invited to take part in the research project during medical visits, after obtaining the prior consent of the director of the facility. Moreover, the participants of the University of the Third Age classes were informed about the conducted research in the form of a poster advertisement. In total, 600 women were invited to the study, and 262 decided to participate. All of them were aware which group they would belong to, after the results of the densitometric test. Before joining the project, each of the women was acquainted with the terms of participation, which are presented in Table 1.

**Table 1:** Inclusion and exclusion criteria.

Inclusion criteria (women with osteoporosis)	Exclusion criteria (both groups)
• Participant's age between 60 and 65, • Diagnosed with osteoporosis • Informed consent of the patient to participate in the study, • State of physical and / or mental fitness allowing participation in the study, • No contraindications to taking up physical exercise, • Fall in an interview—obtaining as detailed information as possible about the circumstances and consequences of the incident	• Lack of informed consent to participate in the study, • Age under 60 and over 65, • The state of physical and / or mental fitness does not allow participation in the study, • Contraindication to exercise, • Significant orthopedic diseases that significantly limit joint mobility, e.g. advanced osteoarthritis, joint stiffness, coxarthrosis, • Rheumatic diseases such as rheumatism, • Neurological diseases such as: Parkinson's disease, post-stroke condition, Huntington's disease, labyrinthine disorders, • Active neoplastic disease

Each participant of the study gave a informed consent, which guaranteed protection of her medical and personal data, as well as the assurance that the results would not be processed other than for scientific purposes. In addition, as part of the project, each of the women could participate in Nordic Walking classes to encourage them to physical activity. The height of the body of patients with osteoporosis was average 1.59 ± 0.05 m, while among non-osteoporotic patients 1.6 ± 0.05 m. The average body weight in the study group was 71.60 ± 14.85 kg, and in the control group 72.75 ± 11.81 kg. Meanwhile, BMI was on average 28.01 ± 5.57 kg/m^2^ in women with osteoporosis and 28.46 ± 4.74 kg/m^2^ in others. The group of women with osteoporosis included mainly married women (63.7%), living in the city (92.6%) with secondary (45.9%) and higher (38.5%) education. On the other hand, the group of healthy women included mainly married women (85.8%) living in the city (77.2%) with higher education (55.1%). Table 2 contains all the basic information characterizing the entire study group. The research was conducted in the period of April 2014 to November 2015.

**Table 2:** Basic characteristic.

Described parameter		The group of women with osteoporosis	The group of healthy women
Age (x̄ ± sd) [years]		63 ± 1.8	62 ± 1.9
Height (x̄ ± sd) [m]		1.59 ± 0.05	1.6 ± 0.05
Weigh (x̄ ± sd) [kg]		71.62 ± 14.85	72.75 ± 11.81
BMI (x̄ ± sd) [kg/m^2^]		28.01 ± 5.57	28.46 ± 4.74
Marital status ( %, N)	Married	63.7% N = 86	85.8% N = 109
	Widow	23.7% N = 32	8.7% N = 11
	Divorcee	9.6% N = 13	4.7% N = 6
	Single	2.9% N = 4	0.8% N = 1
Place of residence	City	92.6% N = 125	77.2% N = 98
	Village	7.4% N = 10	22.8% N = 29
Education	Primary education	4.4% N = 6	6.3% N = 8
	Vocational education	11,1% N = 15	4.7% N = 6
	Secondary education	45.9% N = 62	33.8% N = 43
	Higher education	38.5% N = 52	55.1% N = 70

### Assessment of physical fitness using the senior fitness test (SFT)

The level of physical fitness was assessed by means of the Senior Fitness Test (SFT), which was applied because of the ease of its performance and of result interpretation. The test indirectly assesses the strength of the upper and lower body parts, aerobic performance capacity, suppleness of the upper and lower body parts, motor coordination, and balance. Also, before and after each trial, the patients' blood pressure and pulse were checked. The test was carried out in the methodologically specified order of trials: 30-s Chair Stand Test, 30-s Arm Curl Test, 2 min Walk Test, Chair Sit-and-Reach Test, Back Scratch Test. Each of the tests was performed three times, and the best result was recorded. The 8-Foot Up-and-Go Test was omitted in the research because walking time measurement was completed in the Timed Up and Go Test. Strength of the upper body part was assessed by means of the 30-s Arm Curl Test. The assessment of the lower body part strength was made using the 30-s Chair Stand Test. The chair stand and sit is one full repetition [9. 10]. The upper body part (shoulder) flexibility test was performed in a standing position by means of the Back Scratch Test. When the patient's fingers overlap, the value is positive (" + "), if not, it is negative ("−")^9, 10^. The lower body part (primarily hamstring) flexibility test—the Chair Sit-and-Reach Test. The examined women tried to reach their toes with their palm fingers and maintain that position for 2 s. When palm fingers were crossing the line of the toes, the measurement was considered to be positive (" + "), if not it was negative ("−")^9, 10^. All measurements in suppleness tests were made with the accuracy of 0.5 cm.

Table 3 presents the American SFT standards for women aged 60–65 used in the study. There are currently no other scientifically accepted standards in Europe.

**Table 3:** Standards for SFT tests used in the study (Jones C.J, Rikli R.).

Type of trial	The result—the norm of 60–65 years
30-s chair stand test (no. of stands/30 s)	12–17
30-s arm curl test (no. of reps/30 s)	13–19
2 min walk test–test 2 (no of steps)	75–107
Chair sit-and-reach test (inches + /−)	− 0.5–5.0
Back scratch test (inches +/−)	− 3.0–1.5

### Fall risk assessment

The POMA (Performance-Oriented Mobility Assessment) Tinetti test is a method of a comprehensive assessment of balance and dexterous mobility of the examined person. It is a clinical prophylaxis tool, which is very frequently applied at walking and balance disorders^6^. The POMA Tinetti test applied in this study was composed of two parts. The first one included nine positions, relating to the assessment of balance in sitting, rising, standing, turning, and sitting down. The second part covered seven positions, which were assessing walk considering its initiation, step length and height, step symmetry, walking path, and body position. For each position, the examined woman could score 0, 1, or 2 points, and the maximum score was 28 points. Final result below 26 points indicated the existence of the fall risk, and scoring less than 19 points meant risk that was five-times higher^6, 11^. The test reliability index was ICC = 0.93, sensitivity 64%, and specificity 66% for distinguishing patients with and without a fall^11^.

Timed Up and Go Test (TUG) is a modification of the Get Up and Go Test developed by Podsiadło and Richardson, which consists in replacing the five-grade point assessment with test completion time measurement. This is the simplest form of assessment of walking and balance. The task of the examined person was to rise from a chair (the seat height 46 cm) from a sitting position with her back leaning against the chair backrest, then walk a distance of 3 m in a flat terrain, cross the line bordering on the determined stretch of path, turn around by 180°, return to the chair, and sit down again. The test completion time was measured from the "start" command issued to the person sitting on the chair until the moment of taking up the sitting position again. The examined persons were asked to complete the task as quickly as possible, yet in a pace, which was safe for them^6, 11, 12^. When interpreting the result, the lack of a risk fall was assumed at the time below 10 s, while scores between 10 and 20 s constituted a low risk of fall, with a high risk of fall upon scores exceeding 20 s^6^.

### Statistical analysis

The analysis of test results was carried out using Statistica 12.0 software package and MS Excel 2013. Homogeneity of both groups in terms of age was varied using the Shapiro–Wilk test. Basic statistical parameters were calculated: the arithmetic mean (x̄), standard deviation (sd) and scatter (R_max_-_min_). The level of statistical significance for differences between average results of both groups was determined by means of T-student test. Correlations were determined using the Pearson coefficient. Test probability on levels p < 0.05 was assumed to be statistically significant.

### Informed consent

Informed consent was obtained from all individual participants included in the study. The research was conducted with the consent of the Bioethics Committee at the Medical Chamber in Cracow.

## Results

The majority of women with osteoporosis were ill for about 1 to 3 years (31.1%—under one year; 38.5%—from 1 to 3 years). Most of them found out about the disease during preventive examinations (78.5%). Almost half of women treat osteoporosis only with Ca and vitamin supplementation D3 (42.2%). Treatment is not used by as many as 28.2% of sick women. Less than a third of them are treated with pharmacotherapy prescribed by a doctor (14.1%) enriched with Ca and vitamin D3 supplementation (15.6%). Table 4 presents basic information on the course of osteoporosis among the surveyed women.

**Table 4:** Basic information about osteoporosis among the surveyed women.

How long has she been ill?	< 1 year	31.1% (N = 42)
	1–3 years	14.1% (N = 19)
	4–6 years	38.5% (N = 52)
	> 6 years	16.3% (N = 22)
Type of diagnosis	Preventive examinations	78.5% (N = 106)
	At breakage	11.1% (N = 15)
	Performing densitometry due to being classified as a risk group	8.8% (N = 12)
	Densitometry prescribed by a doctor	0.7% (N = 1)
	While performing an X-ray of the spine	0.7% (N = 1)
Treatment used	Medication prescribed by a doctor	14.1% (N = 19)
	Medication prescribed by a doctor with Ca supplementation	15.5% (N = 21)
	Medication prescribed by a doctor with Ca and Vitamin D_3_ supplementation	42.2% (N = 57)
	Only Ca and Vitamin D_3_ supplementation	14.1% (N = 19)
	Doesn’t apply treatment	28.1% (N = 38)

All women had correctly performed 3 battery tests of the SFT tests. No adverse events were recorded during testing. In the assessment of the lower body part strength, the average number of exercise repetitions over 30 s amounted to 13 ± 4.3 in the GO group, and to 12 ± 3.69 in the GH group. The difference between the two groups was statistically significant on the level of p < 0.05. The scatter of the results obtained in both groups amounted from 6 to 25 repetitions/30 s. The average results of the assessment of the upper limb strength were 14 ± 3.95 repetitions/30 s for the GO group, and 13 ± 2.97 repetitions/30 s for the GH group, respectively. The difference between both groups was not statistically significant. In the GO group, the scatter of the obtained results amounted from 6 to 28 repetitions/30 s, and the GH group had 6 to 21 repetitions/30 s. In the aerobic performance capacity, the average result was 78 ± 20.48 steps/2 min for the GO group, and 83 ± 18.78 steps/2 min for the GH group, respectively. The difference between the groups was not statistically significant. In the GO group the examined women were making 32 to 155 steps/2 min, while in the GH group from 50 to 155 steps/2 min. In the assessment of flexibility of the lower body part, the average trial result in the GO group was 1.72 ± 9.28 cm, and in the GH group 0.82 ± 5.09 cm. The difference between both groups' results was statistically significant on the level of p < 0.05. The range of measurement in the GO group oscillated between − 36 cm and 19 cm, while in the GH group between 9 and 19 cm. In turn, in the assessment of flexibility of the upper body part, the average measurement result in the GO group was − 4.97 ± 9.46 cm, and 0.01 ± 4.12 cm in the GH group. The difference between both groups' results was statistically significant on the level of p < 0.05. Table 5 presents results of the SFT measurements.

**Table 5:** Basic characteristics for senior fitness test batteries in both groups.

Descriptive statistics	The group of women with osteoporosis	The group of healthy woman	p-value for differences between mean values
Strength of the lower body part [reps/30 s]	13.55 ± 4.44	12.53 ± 3.84	p < 0.05
Strength of the upper body part [reps/30 s]	14.09 ± 4.17	13.35 ± 3.11	p > 0.05
Aerobic performance capacity [no. of steps]	78.91 ± 21.70	83.27 ± 19.92	p > 0.05
Flexibility of the lower body part [cm]	-1.54 ± 9.80	0.95 ± 5.43	p < 0.05
Flexibility of the upper body part [cm]	-4.85 ± 9.81	0.14 ± 4.30	p < 0.05

Comparing the obtained measurements with the range of norms, it can be noticed that the strength of the lower body is at a similar level in both groups. More women from the healthy group scored below normal, while more women from the osteoporosis group scored above normal. Thus, in the case of lower body strength assessment, women with osteoporosis fared better. The same situation was noted in the assessment of the upper body. On the other hand, in terms of aerobic fitness, healthy women achieved better results—more of them obtained normal and above normal results, and less below normal. However, in the assessment of the flexibility of the lower body, more healthy women scored within the normal range, but more women with osteoporosis scored above the norm and below the norm. The assessment of upper body flexibility showed a significantly higher level among healthy women in all three aspects—lower number of women with a score below normal, higher in normal and higher above normal. Table 6 depicts comparison of both groups.

**Table 6:** Assessment of SFT physical fitness level.

Parameters assessed	Below the standard		Within the standard		Above the standard	
	GO	GH	GO	GH	GO	GH
Assessment of the lower body part strength	37.0%* (N = 50)	46.3%* (N = 59)	45.9% (N = 62)	45.5% (N = 58)	17.0%* (N = 23)	8.1%* (N = 10)
Assessment of the upper body part strength	44.4% (N = 60)	48.8% (N = 62)	43.0% (N = 58)	45.5% (N = 58)	12.6%* (N = 17)	5.7%* (N = 7)
Assessment of the aerobic performance capacity	40.0%* (N = 54)	30.9%* (N = 39)	54.1%* (N = 73)	62.6%* (N = 79)	5.9% (N = 8)	6.5% (N = 9)
Assessment of the lower body part flexibility	38.5%* (N = 52)	27.6%* (N = 35)	40.7%* (N = 55)	54.5%* (N = 69)	20.7% (N = 28)	17.9% (N = 23)
Assessment of the upper body part flexibility	48.8%* (N = 66)	19.5%* (N = 25)	23.7%* (N = 32)	50.4%* (N = 64)	27.5% (N = 37)	30.1% (N = 38)

(GO – the group of women with osteoporosis, GH – the group of healthy women; * p < *0.05).*

The POMA Tinetti test was reviewed in the parts concerning balance, walking, as well as considered comprehensively. In the assessment of their balance, women from the GO group scored the average result of 14.3 ± 1.78 points, while those from the GH group 14.39 ± 1.6 points, respectively. The difference between both groups' results was not statistically significant. The range of results that were obtained in the GO group fell into a 9–16 points interval, and in the GH group that interval ranged from 11 to 16 points. In the assessment of walking, women from the GO group obtained average results on the level of 11.05 ± 1.62 points, and the GH women 11.5 ± 0.72 points. The difference between both groups' results was also not statistically significant. In the GO group, the range of results fell into a 4–12 points interval, as compared with the interval of 10 to 12 points of the GH group. Total average POMA Tinetti test result in the GO group was 25.26 ± 3.15 points, and 25.88 ± 1.91 points in the GH group. The range of results in the GO group was 14 to 28 points, while the GH group noted the range of 21 to 28 points. In the assessment of the fall risk made with the TUG test, the average result scored in the GO group was 9.24 ± 3.56 s, and 8.14 ± 1.91 s in the GH group. Statistical significance between the results of both groups was recorded on the level of p < 0.05. Trial times obtained in the GO group fell into an interval of 4.4 and 22.95 s. In the GH group the interval was 4.8 and 16 s (Table 7).

**Table 7:** Tinetti POMA test & TUG results.

Descriptive statistics	The group of women with osteoporosis	The group of healthy women	p-value for differences between mean values
Tinetti balance[points]	14.3 ± 1.78	14.39 ± 1.6	p > 0.05
Tinetti walk [points]	11.05 ± 1.62	11.50 ± 0.72	p > 0.05
Tinetti POMA total [points]	25.26 ± 3.15	25.88 ± 1.91	p > 0.05
TUG Test [s]	9.24 ± 3.56	8.14 ± 1.91	p < 0.05 *

(* p < *0.05).*

The correlations between the SFT results and the tests assessing the risk of falling turned out to be weak (below 0.4), despite their statistical significance. Thus, the relationship between the level of physical fitness and the risk of falling cannot be unequivocally demonstrated. However, the highest correlations, close to moderate, were related to strength and aerobic capacity. Correlations described in this place are shown in Table 8.

**Table 8:** Correlations between SFT and Tinetti and TUG tests.

Functional tests	Tinetti balance [points]		Tinetti walk [points]		Tinetti POMA in total [points]		Test TUG [s]	
	GO	GH	GO	GH	GO	GH	GO	GH
Strength of the upper body part [repetitions/30 s]	0.359*	0.313*	**0.258**	0.063	0.343*	0.278*	− 0.071	− 0.044
Strength of the lower body part [repetitions/30 s]	0.385*	0.347*	**0.308**	0.105	0.395*	0.321*	− 0.151	− 0.143
Aerobic performance capacity [number of steps]	0.340*	0.304*	0.295*	0.264*	0.395*	0.347*	− 0.333*	− 0.313*
Suppleness of the upper body part [cm]	**0.180**	0.137	0.085	0.097	0.094	0.148	− 0.117	− 0.137
Suppleness of the lower body part [cm]	0.198*	0.137	0.184*	− 0.103	0.163	0.072	− 0.241*	− 0.300*

(GO—the group of women with osteoporosis, GH—the group of healthy women; * p < *0.05).*

## Discussion

Taking into account the obtained results, it can be concluded that women between 60 and 65 years of age do not show great tendency to fall. There was a significant difference between the two groups only in terms of the TUG test. This may be due to the fact that they are still economically active and the involutionary changes related to aging are not as intense yet. The results of the assessment of physical fitness also showed that the majority of the examined women in both groups achieve normal measurements. Only in terms of assessing the flexibility of the upper and lower body, it was observed that the results of women with osteoporosis were significantly worse compared to those of the healthy ones. It is worth noting here that from the physiotherapeutic point of view, reduced flexibility is associated with a decrease in the flexibility of muscles, ligamentous and joint apparatus. As a result, this will lead to bone formation disorders as well as static and postural disturbances. Thus, the risk of falling due to decreasing physical and functional fitness will begin to increase. Therefore, it seems that the patients from the study group are at the most appropriate age to undertake comprehensive preventive measures, both among patients with osteoporosis and healthy people. It also seems that the disease itself in the form of osteoporosis, in this study, is not a significant determinant of lowering the level of physical fitness and high risk of falling. High level of physical activity is recommended already to women in the premenopausal period, who are in the risk group. Physical activity on a level higher than the current one is recommended for adult women in the premenopausal period. This is connected with improving the quality of the body composition components, and general functional fitness^13^. The aerobic exercises, supported with omega-3 acids supplementation proved to be an efficient activity to undertake in reducing chronic inflammation, and increasing the BMD rate in postmenopausal women suffering from osteoporosis^14^.

Gowin et al.^12^ noted that often the moment, in which osteoporosis has been diagnosed, constitutes such a moment in women's life, following which consequences resulting from the disease begin to affect limitations of those women in their daily functioning. Own research also indicates that considerable lowering of physical fitness causes a deterioration of functional fitness which, in hindsight, will also lead to dependence on third persons. It seems that the critical age of 60 years of age is such a moment, in which stimulation with a healthy lifestyle that includes an increased physical activity may lead to the improvement of quality of life in a later age. It has been demonstrated that exercises aimed at reducing the body mass (in obese women), as well as weight training are indicated for maintaining correct osteogenesis processes and reducing falls and ostpeorotic fractures. In his research, Senter et al.^13^ demonstrated that overloading with a physical effort of 24 METs/hour during a week has reduced the risk of fracture by 55%. Smith et al.^14^ indicate that exercises, which stretch the spine muscles, may increase the risk of compression fractures, yet doing a combination of bends and extensions in the median range of motion may bring benefits in the form of reducing spine pain complaints in women with osteoporosis. Still the authors suggest that each yoga practice must be individually suited, depending on the risk of osteoporotic fracture and tendency to falls.

To women with osteoporosis, a fracture constitutes a very serious social problem. Despite that, to many of them benefits from treatment in the form of fracture and fall prevention are little, and not worth undertaking^3^. Own research shows that women aged 60–65 years of age and suffering from osteoporosis did not demonstrate any major risk of fall both in the assessment with the Tinetti POMA test, and with the TUG test. However, every fourth woman with osteoporosis demonstrated high tendency to falls which, to a large extent, was connected with her lowered physical fitness. The examined age interval of women does not show differences between them yet but may also constitute the last moment for the application, with high effectiveness, of counter-fall prophylaxis. Jeon et al. conducted control, randomised examinations to assess the risk of fall under the impact of a 12-week training programme. First measurements did not show any significant differences between the groups as both of them were marked by a high risk of fall. Yet after 12 weeks of training, considerable differences between the experimental group and the control group were identified, especially in terms of assessment of the muscle strength and endurance. On the other hand, the training has not affected their static balance. The authors show that the programme they are offering constitutes not only a full counter-fall prophylaxis for people with osteoporosis but also constitutes a complete therapeutic training, which develops muscle strength, endurance, balance, and improvement of psychological aspects^15^.

It is worth noting the extensive analysis made by Giangregorio et. al.^16^ about exercise recommendations for individuals with osteoporosis or osteoporotic vertebral fracture. The authors indicate that there were only four trials which examined the effects of exercise on mobility among individuals with osteoporotic vertebral fracture. One of them revealed a small, but significant difference between groups in favor of exercise for Timed Up and Go test performance. The panel strongly recommends a multicomponent exercise program that includes progressive resistance training for all major muscle groups, at least twice a week. It is also strongly recommended that individuals with osteoporosis or osteoporotic vertebral fracture participate in daily balance training, as part of a multicomponent exercise program, and aim to accumulate 2 h of balance training weekly, where daily training can be performed all at once, in short bouts throughout the day, or incorporated into daily activities. Exercises which provide a sufficient challenge to balance should be chosen, by reducing the person’s base of support or amount of sensory input, perturbing their center of mass or challenging muscles important for posture or balance. They may need to be individually tailored^16^.

Taking into account own research, it is possible to say that the effectiveness of proposed training programmes may be higher if one should start programming them based on individual diagnostics that evaluates actual functional and fitness deficits.

Research conducted by Turkish scholars Tüzün et al.^17^ on the impact of yoga on balance and quality of life of women with osteoporosis in the postmenopausal period prove that in comparison to traditional treatment, yoga significantly contributes to reducing pain ailments, and improves functionality of examined women. In addition, yoga positively affected sociological aspects of functioning of sick women. Research demonstrated a statistically significant improvement as regards balance and general health condition. Based on all results obtained, the authors indicate some exercise of yoga could be a good alternative in osteoporosis rehabilitation. According to McArthur et al.^18^ Yoga can be included in a multicomponent exercise program if consideration is given to which postures are safe and how to transition into and out of them. What is more general guidelines for exercise is that older adults with osteoporosis should participate in a multicomponent exercise program, including resistance and balance training. Contraindicated movements include end-range flexion/extension/rotation of the spine and internal/external rotation of the hip. Yoga postures that should be encouraged include postures emphasizing spinal alignment and extension to mid-range in standing and on the floor^18^.

It seems necessary to examine women patients with osteoporosis with a fully functional and fitness-oriented approach to eliminate, and/or reduce the risk of fall and its consequences.

The presented study was a pilot study and its main aim was to provide empirical data to improve knowledge on the level of physical fitness and mobility among young elderly women suffering from osteoporosis. As the results were processed, the authors observed gaps in their management, especially in collecting additional medical information on past fractures and injuries. It was probably due to the fact, among other reasons, that the problem under investigation interested us more from a physiotherapeutic and physioprophylactic point of view. Nevertheless, in further research, we primarily intend to broaden the research sample and collect additional medical information.

## Conclusions

The level of physical fitness of women with osteoporosis and healthy women is very similar, in terms of flexibility of the upper and lower body, women suffering from osteoporosis obtained much worse results. Moreover, it turned out that women in the examined age group do not show a high tendency to fall. The performed analysis did not show any significant correlation between the level of physical fitness and the risk of falling among the surveyed women.
